# Adult-Onset Hemophagocytic Lymphohistiocytosis Associated With Epstein-Barr Virus, Lyme Disease, and Anaplasmosis

**DOI:** 10.7759/cureus.72575

**Published:** 2024-10-28

**Authors:** Salman Syed, Girish Shah, Garry Lachhar, Neel Patel, Farhad Mohammad Amjad

**Affiliations:** 1 Internal Medicine, Northwell Health, New York, USA; 2 Hematology and Oncology, Northwell Health, New York, USA

**Keywords:** anaplasmosis, ebv, hlh, lyme, sepsis

## Abstract

Adult-onset hemophagocytic lymphohistiocytosis (HLH) is a life-threatening hyperinflammatory syndrome characterized by dysregulated immune activation. Diagnosing HLH poses significant challenges due to its nonspecific clinical presentation, which often mimics infections, malignancies, and autoimmune diseases. Early recognition and prompt initiation of immunosuppressive therapy are crucial, as delayed treatment is associated with a high risk of mortality.

We present a unique case of HLH triggered by a combination of Epstein-Barr virus (EBV), Lyme disease, and anaplasmosis, underscoring the complexity of diagnosing and managing this condition. The patient initially presented with fever, hypotension, and altered mental status, raising concerns for septic shock. However, a comprehensive diagnostic workup, including infectious disease testing, hematologic evaluation, and bone marrow biopsy, revealed HLH with evidence of EBV viremia, *Borrelia burgdorferi* exposure, and anaplasmosis infection.

This case highlights the importance of maintaining a broad differential diagnosis when confronted with a sepsis-like presentation, particularly in endemic regions where tick-borne diseases and viral infections coexist. A multidisciplinary approach involving infectious disease specialists, hematologists, and intensivists was essential in achieving the diagnosis and formulating a treatment plan. The patient responded favorably to immunosuppressive therapy, including corticosteroids, intravenous immunoglobulins, and targeted antimicrobial therapy, resulting in clinical stabilization and recovery.

Our report emphasizes the need for heightened clinical awareness of HLH and the challenges posed by its overlapping features with other systemic illnesses. Furthermore, it illustrates the significance of early intervention and individualized care in managing HLH triggered by multiple infectious agents.

## Introduction

Hemophagocytic lymphohistiocytosis (HLH) is a rare but life-threatening hyperinflammatory syndrome characterized by excessive immune activation and cytokine release [1]. Familial/primary HLH is common in children and is caused by genetic mutations. Secondary HLH is precipitated by various triggers, including viral infections such as Epstein-Barr virus (EBV), HIV, and cytomegalovirus (CMV), as well as bacterial, parasitic, and fungal organisms [1]. Malignancies, particularly lymphoma, autoimmune conditions, and other factors like organ transplantation, surgery, or hemodialysis, can also serve as triggers [2]. Both primary and secondary HLH are characterized by elevated levels of ferritin and inflammation, sharing a common terminal pathway despite differing pathogenic origins [3]. HLH represents an abnormal immune reaction propelled by T cells and is associated with potentially life-threatening cytokine storms [4].

This report focuses on an unusual case of HLH precipitated by co-infection with EBV, CMV, and anaplasmosis.

HLH presents with a constellation of clinical features such as prolonged fever, hepatosplenomegaly, cytopenias, and hyperferritinemia, making early diagnosis challenging due to its overlap with other conditions. The co-infection with EBV, CMV, and anaplasmosis is particularly unique, as each pathogen can independently trigger HLH. However, their concurrent presence may exacerbate the clinical severity and complicate management strategies.

This case underscores the importance of considering HLH in the differential diagnosis when a patient presents with persistent fevers and cytopenias, especially in the context of known infectious triggers. It also highlights the need for a multidisciplinary approach to effectively identify and manage underlying infections.

## Case presentation

**Figure 1:**
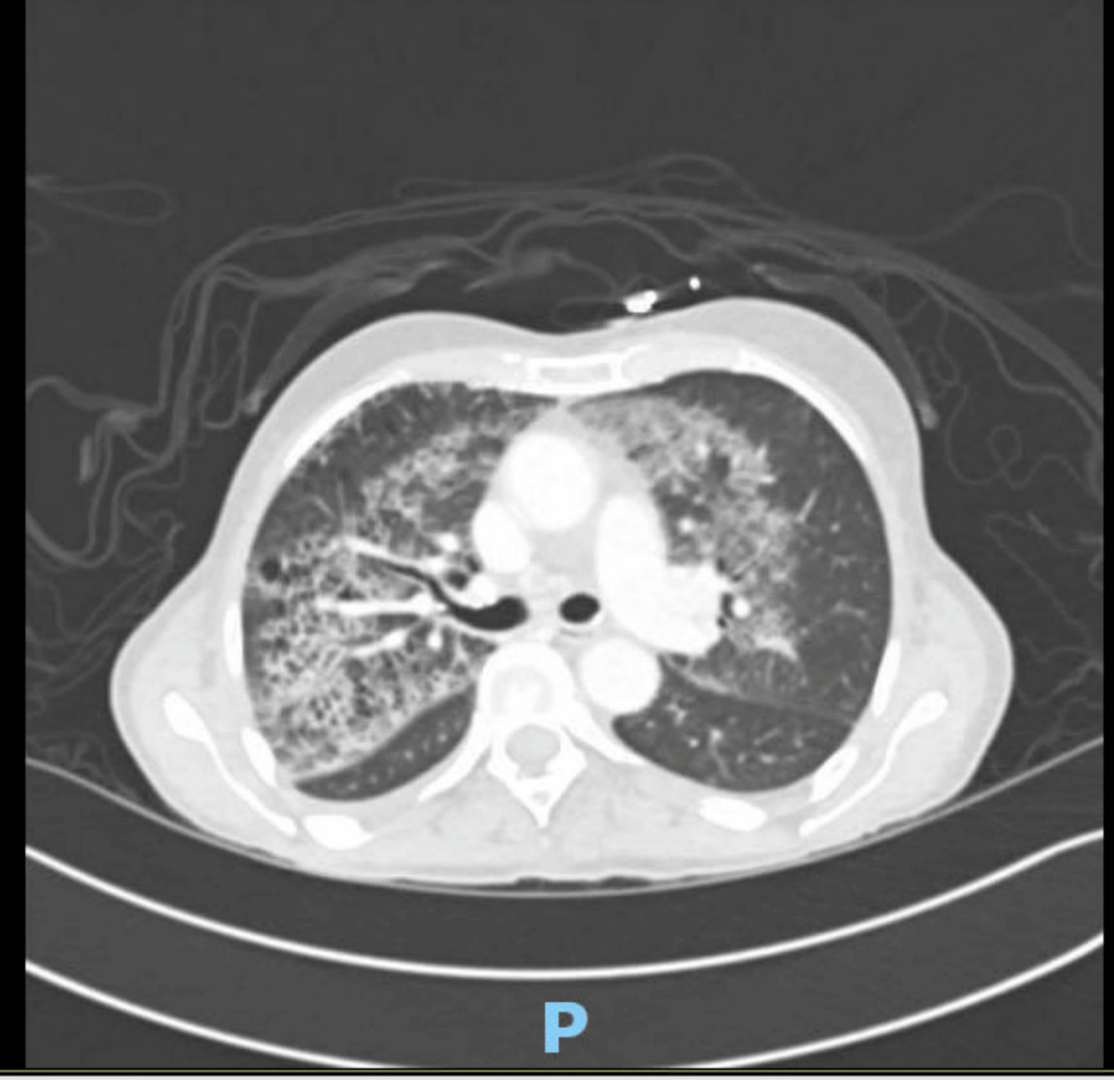
Shows bilateral pneumonia.

We have a 68-year-old Caucasian female resident of the tri-state area with a complex medical history. Thirteen years ago, she underwent surgery, chemotherapy, and radiotherapy for breast cancer. She also has a history of chronic obstructive pulmonary disease (COPD) and has been a smoker for forty pack-years. She has no recent travel history. Despite her significant medical background, she was not actively receiving any medication at the time of presentation and presented with weakness and malaise. The patient was found to be febrile to 103.2°F, tachypneic, tachycardic, and hypotensive. Laboratory analyses revealed bicytopenia with a WBC count of 2.68 cells/microliter and neutrophil bands of 11.4%, and platelet counts on presentation were 28K. Her hemoglobin was 12.8, and initial troponins were 21>8>6 (usually less than 14 mg/L). Serum sodium was 129, and plasma osmolarity was 275. Additionally, abnormal liver function tests included aspartate aminotransferase (AST) 155 and alanine transaminase/serum glutamic pyruvic-transaminase (ALT/SGPT) 42. Alkaline phosphatase and bilirubin were within normal limits at 63 and 0.6, respectively. Her kidney functions, including blood urea nitrogen and creatinine, were 23 and 0.7. Her procalcitonin was elevated up to 1.11 mg/dl; urinalysis indicated the presence of leukocytes and a positive culture screening for which she was later treated. While a chest X-ray exhibited no acute cardiopulmonary conditions, the patient was admitted with the diagnosis of sepsis due to a UTI and was placed on ceftriaxone 1 g daily. On the floor, the patient’s clinical condition started worsening, including a drop in blood pressure to 76/42 mmHg, a fever of 104.2°F, and worsening leukopenia from 2.48 to 1.14 K/ul; a subsequent CT scan revealed bilateral pneumonia (Figure 1).

Notably, the respiratory panel revealed no evidence of a viral etiology. Another CT scan focusing on the abdomen showed no signs of lymphadenopathy, splenomegaly, or hepatomegaly. Given the above clinical setting, the patient was placed on treatment for community-acquired pneumonia, which did not require any oxygen. The ICU was consulted due to hemodynamic instability. She was transferred to the ICU for septic shock and was placed on cefipime, vancomycin, and doxycycline. Further investigations demonstrated abnormal coagulation parameters and elevated ferritin. Her hemoglobin remained stable, but her lactate dehydrogenase was 2125, and her haptoglobin was less than 20 mg/dl. Her ferritin was elevated up to 100,000 ng/dl, and repeat lab values showed 47656 ng/dl with no clinical signs of iron overload. Her Coombs' test was negative to rule out hemolytic anemia. Her coagulation parameters were normal for prothrombin time (PT) at 11.3 sec and INR at 1.03 sec. Her activated partial thromboplastin time (APTT) was 48.8 s, and her D-dimers were 3355. Her fibrinogen level of 92 raised the suspicion of disseminated intravascular coagulation, and the patient received two units of cryoprecipitate (Table 1).

**Table 1:** Lab values. AST: Aspartate aminotransferase; ALT: Alanine transaminase; SGPT: Serum glutamic pyruvic transaminase; BUN: Blood Urea Nitrogen; PT: Prothrombin Time; INR: International Normalized Ration; APTT: Activated Partial Thromboplastin Time.

Lab Test	Reference Range	Patient's Lab values
WBC Count	3.8-10.5 K/μL	2.68
Bands	0-8%	11.4
Platelet Count	150-450 K/μL	28
Serum Sodium	136-145 mmol/L	129
Plasma Osmolarity	280-300 mOsm/kg	275
AST	0-32 U/L	155
ALT/SGPT	0-33 U/L	42
Alkaline Phosphatase	35-104 U/L	63
Bilirubin	0-1.2 mg/dL	0.6
BUN	8-23 mg/dL	23
Creatinine (Cr)	0.7-1.2 mg/dL	0.7
Procalcitonin	<0.5 ng/mL	1.11
Lactate Dehydrogenase	134-214 U/L	2125
Haptoglobin	30-200 mg/dL	20
Ferritin	13-150 ng/mL	100,000
PT	10.5-12.8 seconds	11.3
INR	0.9-1.12	1.03
APTT	25.5-36.5 seconds	49
D-Dimers	<230 ng/dL	3355
Fibrinogen	193-461 mg/dL	92
Triglycerides	<150 mg/dL	234

Meanwhile, her triglyceride level was 234 mg/dl. Her Lyme disease serology, including IgG and IgM, came out to be positive, along with EBV and anaplasmosis, while negative for babesiosis and Ehrlichia. Later, PCR was positive for Lyme, anaplasmosis, and EBV. Her suspicion of HLH was increased, and an H-score was calculated, which without a bone marrow biopsy was 234 points, indicating a 98-99% probability. 

The patient was treated with IV immunoglobulins and dexamethasone.

The patient positively responded to the treatment, and a subsequent bone marrow biopsy revealed significant pathological findings consistent with HLH. Upon discharge, the patient was prescribed oral steroids, doxycycline, and trimethoprim-sulfamethoxazole for prophylaxis against *Pneumocystis jiroveci* pneumonia. Continued follow-up with the hematology, oncology, and infectious disease specialists was strongly advised to ensure comprehensive long-term management.

## Discussion

HLH is a highly aggressive and life-threatening syndrome of excessive immune activation. In adults, secondary HLH often develops in association with infections, malignancies, or autoimmune diseases, leading to severe hyperinflammation [5-7]. The case presented here is particularly notable due to the involvement of multiple infectious agents, EBV, Lyme disease, and anaplasmosis, all of which can independently trigger HLH, making this a rare and complex clinical scenario.

The patient's presentation, characterized by fever, cytopenia, and multiorgan dysfunction, initially mimicked sepsis. This overlap in clinical features with other critical conditions, such as sepsis and disseminated intravascular coagulation (DIC), complicates the early diagnosis of HLH. The diagnostic challenge is further amplified by the rarity of HLH in adults, making it a less likely consideration in the initial differential diagnosis. However, despite broad-spectrum antibiotic coverage, the rapid deterioration of the patient's clinical status prompted a deeper investigation into potential underlying causes of immune dysregulation [8,9].

Several vital findings supported the diagnosis of HLH, including hyperferritinemia, elevated lactate dehydrogenase (LDH), low fibrinogen levels, and a positive H-score indicating a high probability of HLH. The detection of multiple infections, particularly EBV, a well-known trigger for HLH, alongside positive serology and PCR for Lyme disease and anaplasmosis, further complicated the clinical picture. The simultaneous presence of these infections likely contributed to the severe immune activation observed in this patient.

This case highlights the importance of a multidisciplinary approach in managing patients with suspected HLH. Early involvement of hematology, infectious disease, and critical care teams was crucial in arriving at the correct diagnosis and initiating appropriate treatment. The use of intravenous immunoglobulins (IVIG) and dexamethasone, part of the HLH treatment protocol, proved effective in controlling the hyperinflammatory response and improving the patient’s clinical condition.

Furthermore, this case underscores the importance of considering HLH in patients with unexplained cytopenias and systemic inflammatory responses, especially when common causes such as sepsis have been ruled out or when there is a lack of clinical improvement despite appropriate treatment. The co-infection with EBV, Lyme disease, and anaplasmosis in this patient serves as a reminder of the complex interplay between infections and immune dysregulation in the pathogenesis of HLH.

## Conclusions

This case report illustrates the critical importance of a thorough diagnostic approach in managing adult-onset HLH, particularly when triggered by multiple concurrent infections such as EBV, Lyme disease, and anaplasmosis. The patient's presentation, initially resembling sepsis, underscores the diagnostic difficulties inherent in HLH, where the syndrome's rarity and overlapping clinical features with other conditions often delay diagnosis and treatment.

The successful management of this case, involving the prompt use of IVIG and dexamethasone, demonstrates that with early recognition and appropriate intervention, even severe cases of HLH can be effectively treated. This case also highlights the value of a multidisciplinary approach in complex cases, where expertise from various specialties is essential in guiding diagnosis and therapy.

In conclusion, clinicians should maintain a high index of suspicion for HLH in patients with persistent fever, cytopenias, and multiorgan involvement, especially when common infectious or inflammatory conditions fail to explain the clinical picture. This case contributes to the growing body of literature on adult-onset HLH and emphasizes the need for continued awareness and research into the triggers, diagnosis, and management of this rare but deadly syndrome.
